# 2-Cyano-*N*′-[(*E*)-1-(2-oxo-2*H*-chromen-3-yl)ethyl­idene]acetohydrazide

**DOI:** 10.1107/S1600536812019915

**Published:** 2012-06-13

**Authors:** Samina Khan Yusufzai, Hasnah Osman, Habibah A. Wahab, Mohd Mustaqim Rosli, Ibrahim Abdul Razak

**Affiliations:** a School of Chemical Sciences, Universiti Sains Malaysia, 11800 USM, Penang, Malaysia; b Pharmaceutical Design and Simulation (PhDS) Laboratory, School of Pharmaceutical Sciences, Universiti Sains Malaysia, 11800 Minden, Pulau Pinang, Malaysia; c X-ray Crystallography Unit, School of Physics, Universiti Sains Malaysia, 11800 USM, Penang, Malaysia

## Abstract

In the title compound, C_14_H_11_N_3_O_3_, the chromene ring is almost planar, with a maximum deviation of 0.065 (2) Å from the mean plane for one of the C atoms. In the crystal, inversion dimers linked by pairs of N—H⋯O hydrogen bonds generate *R*_2_^2^(8) loops. The dimers are linked by C—H⋯N and C—H⋯O inter­actions into a three-dimensional network. An aromatic π–π stacking inter­action, with a centroid–centroid distance of 3.562 (10) Å, is also observed.

## Related literature

For related structures and background to coumarin, see: Yusufzai, Osman, Sulaiman *et al.* (2012 ▶); Yusufzai, Osman, Abdul Rahim *et al.* (2012 ▶).
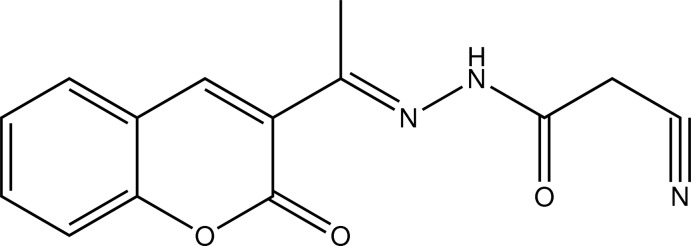


## Experimental

### 

#### Crystal data

C_14_H_11_N_3_O_3_
*M* *_r_* = 269.26
Monoclinic, 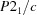

*a* = 10.4755 (2) Å
*b* = 15.8283 (3) Å
*c* = 8.2650 (2) Å
β = 106.982 (2)°
*V* = 1310.66 (5) Å^3^
*Z* = 4
Mo *K*α radiation
μ = 0.10 mm^−1^
*T* = 100 K
0.20 × 0.18 × 0.13 mm

#### Data collection

Bruker SMART APEXII CCD diffractometer
Absorption correction: multi-scan (*SADABS*; Bruker, 2009 ▶) *T*_min_ = 0.980, *T*_max_ = 0.988
13242 measured reflections
3020 independent reflections
1987 reflections with *I* > 2σ(*I*)
*R*_int_ = 0.061

#### Refinement

*R*[*F* ^2^ > 2σ(*F* ^2^)] = 0.057
*wR*(*F* ^2^) = 0.120
*S* = 1.03
3020 reflections
225 parameters
All H-atom parameters refined
Δρ_max_ = 0.27 e Å^−3^
Δρ_min_ = −0.26 e Å^−3^

### 

Data collection: *APEX2* (Bruker, 2009 ▶); cell refinement: *SAINT* (Bruker, 2009 ▶); data reduction: *SAINT*; program(s) used to solve structure: *SHELXTL* (Sheldrick, 2008 ▶); program(s) used to refine structure: *SHELXTL*; molecular graphics: *SHELXTL*; software used to prepare material for publication: *SHELXTL* and *PLATON* (Spek, 2009 ▶).

## Supplementary Material

Crystal structure: contains datablock(s) I, global. DOI: 10.1107/S1600536812019915/hb6757sup1.cif

Structure factors: contains datablock(s) I. DOI: 10.1107/S1600536812019915/hb6757Isup2.hkl

Supplementary material file. DOI: 10.1107/S1600536812019915/hb6757Isup3.cml

Additional supplementary materials:  crystallographic information; 3D view; checkCIF report

## Figures and Tables

**Table 1:** Hydrogen-bond geometry (Å, °)

*D*—H⋯*A*	*D*—H	H⋯*A*	*D*⋯*A*	*D*—H⋯*A*
N2—H1*N*2⋯O3^i^	0.96 (2)	1.91 (2)	2.870 (2)	174 (2)
C3—H3*A*⋯N3^ii^	1.00 (2)	2.53 (2)	3.446 (3)	152.9 (12)
C4—H4*A*⋯N3^iii^	0.96 (2)	2.62 (2)	3.404 (3)	139.2 (16)
C6—H6*A*⋯O2^iv^	0.96 (2)	2.56 (2)	3.494 (3)	164.7 (17)
C13—H13*A*⋯O2^v^	0.96 (2)	2.38 (2)	3.328 (3)	171.7 (18)
C13—H13*B*⋯N3^vi^	1.00 (2)	2.46 (2)	3.409 (3)	159.3 (18)

Symmetry codes: (i) 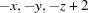; (ii) 
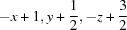
; (iii) 
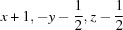
; (iv) 
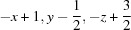
; (v) 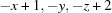; (vi) 
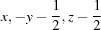
.

**Fig. 1.**
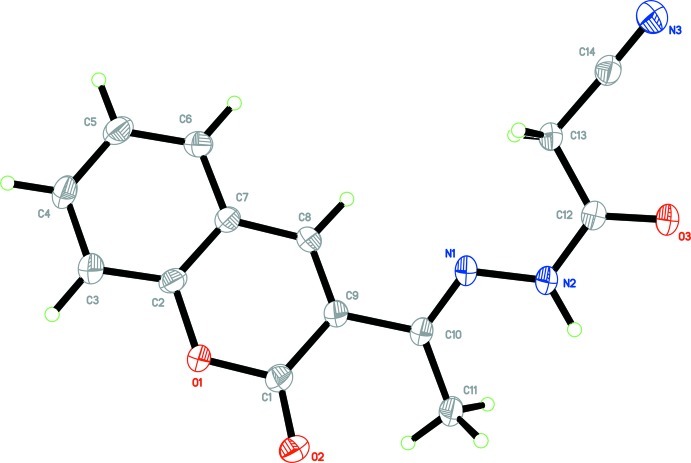
Thermal ellipsoid plot.

**Fig. 2.**
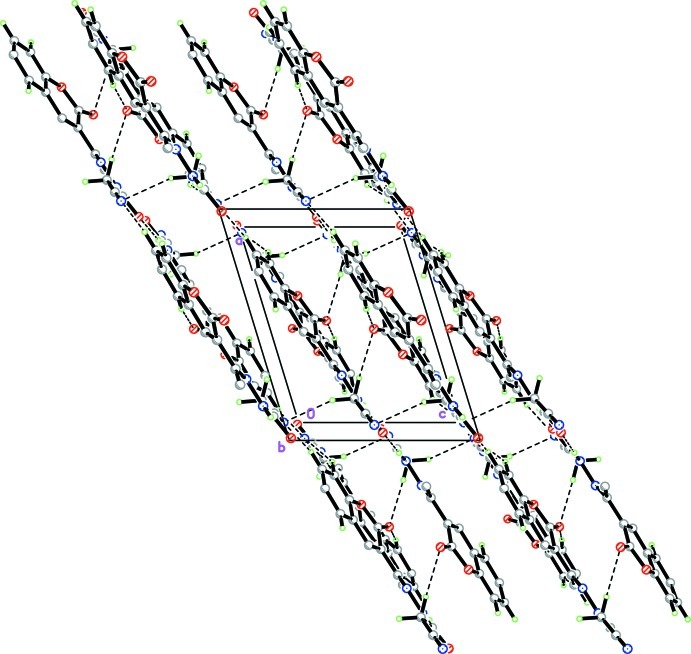
Packing diagram.

## supplementary crystallographic information

### Comment

In continuation of our previous work of coumarin derivatives (Yusufzai, Osman, Sulaiman *et al.*, 2012; Yusufzai, Osman, Abdul Rahim *et al.*, 2012) we have synthesized the title compound: its melting point found to be 175–178°C. Synthesis of other derivatives of coumarin cyanoacetohydrazone and their biological activities are under progress.

The chromene ring is almost planar with the maximum deviation of 0.065 (2) Å from atom C1. In the crystal structure, N2—H1N2···O3^i^, C3—H3A···N3^ii^, C4—H4A···N3^iii^ and C6—H6A···O2 interactions link the molecules into layers parallel to the (1 0 2) plane (Table 1, Fig. 2). These layers are further connected by C13—H13A···O2^v^ and C13—H13B···N3^vi^ intermolecular interactions to form a three-dimensional network (Table 1, Fig. 2). A π—π interaction with centroid-centroid distance of 3.562 (10) Å is also observed (*Cg*1 = O1/C1—C2/C7—C9, *Cg*2 = C2—C7, 1 - *x*, -*y*,1 - *z*).

### Experimental

To a solution of 3-acetyl-2*H*-chromen-2-one. (0.188 g, 0.001 mol) in methanol (20 ml), cyanoacetic acid hydrazide (0.98 g m, 0.001 mol) was added with stirring at room temperature. Hydrochloric acid (0.5 ml) was added and the reaction mixture was stirred at 5–10 ° C for 30 min. The solid product thus formed was collected by filtration, dried in vacuum and recrystallized from ethanol-dioxane (2:1) solution to give the title compound as shiny light yellow blocks.

### Refinement

All H atoms were located in a difference Fourier map and freely refined.

### Crystal data

**Table:**

C_14_H_11_N_3_O_3_	*F*(000) = 560
*M**_r_* = 269.26	*D*_x_ = 1.365 Mg m^−^^3^
Monoclinic, *P*2_1_/*c*	Mo *K*α radiation, λ = 0.71073 Å
Hall symbol: -P 2ybc	Cell parameters from 1883 reflections
*a* = 10.4755 (2) Å	θ = 2.9–32.3°
*b* = 15.8283 (3) Å	µ = 0.10 mm^−^^1^
*c* = 8.2650 (2) Å	*T* = 100 K
β = 106.982 (2)°	Block, yellow
*V* = 1310.66 (5) Å^3^	0.20 × 0.18 × 0.13 mm
*Z* = 4	

### Data collection

**Table:**

Bruker SMART APEXII CCD diffractometer	3020 independent reflections
Radiation source: fine-focus sealed tube	1987 reflections with *I* > 2σ(*I*)
Graphite monochromator	*R*_int_ = 0.061
φ and ω scans	θ_max_ = 27.5°, θ_min_ = 2.0°
Absorption correction: multi-scan (*SADABS*; Bruker, 2009)	*h* = −13→11
*T*_min_ = 0.980, *T*_max_ = 0.988	*k* = −20→20
13242 measured reflections	*l* = −10→10

### Refinement

**Table:**

Refinement on *F*^2^	Primary atom site location: structure-invariant direct methods
Least-squares matrix: full	Secondary atom site location: difference Fourier map
*R*[*F*^2^ > 2σ(*F*^2^)] = 0.057	Hydrogen site location: inferred from neighbouring sites
*wR*(*F*^2^) = 0.120	All H-atom parameters refined
*S* = 1.03	*w* = 1/[σ^2^(*F*_o_^2^) + (0.0477*P*)^2^ + 0.221*P*] where *P* = (*F*_o_^2^ + 2*F*_c_^2^)/3
3020 reflections	(Δ/σ)_max_ < 0.001
225 parameters	Δρ_max_ = 0.27 e Å^−^^3^
0 restraints	Δρ_min_ = −0.26 e Å^−^^3^

### Special details

**Table:**

Experimental. The crystal was placed in the cold stream of an Oxford Cryosystems Cobra open-flow nitrogen cryostat (Cosier & Glazer, 1986) operating at 100.0 (1) K.
Geometry. All e.s.d.'s (except the e.s.d. in the dihedral angle between two l.s. planes) are estimated using the full covariance matrix. The cell e.s.d.'s are taken into account individually in the estimation of e.s.d.'s in distances, angles and torsion angles; correlations between e.s.d.'s in cell parameters are only used when they are defined by crystal symmetry. An approximate (isotropic) treatment of cell e.s.d.'s is used for estimating e.s.d.'s involving l.s. planes.
Refinement. Refinement of *F*^2^ against ALL reflections. The weighted *R*-factor *wR* and goodness of fit *S* are based on *F*^2^, conventional *R*-factors *R* are based on *F*, with *F* set to zero for negative *F*^2^. The threshold expression of *F*^2^ > σ(*F*^2^) is used only for calculating *R*-factors(gt) *etc*. and is not relevant to the choice of reflections for refinement. *R*-factors based on *F*^2^ are statistically about twice as large as those based on *F*, and *R*- factors based on ALL data will be even larger.

### Fractional atomic coordinates and isotropic or equivalent isotropic displacement parameters (Å^2^)

**Table:**

	*x*	*y*	*z*	*U*_iso_*/*U*_eq_	
O1	0.63816 (12)	0.09170 (8)	0.75003 (16)	0.0247 (3)	
O2	0.51923 (13)	0.17039 (9)	0.87086 (18)	0.0315 (4)	
O3	0.00031 (13)	−0.10885 (9)	1.00205 (17)	0.0299 (4)	
N1	0.26272 (15)	−0.03027 (11)	0.86808 (19)	0.0240 (4)	
N2	0.14627 (15)	−0.02929 (11)	0.9143 (2)	0.0248 (4)	
N3	0.05268 (18)	−0.31915 (12)	0.9689 (2)	0.0381 (5)	
C1	0.52801 (19)	0.10271 (13)	0.8072 (2)	0.0244 (4)	
C2	0.67126 (19)	0.01490 (12)	0.6931 (2)	0.0225 (4)	
C3	0.7888 (2)	0.01131 (14)	0.6498 (2)	0.0266 (5)	
C4	0.8249 (2)	−0.06527 (14)	0.5953 (2)	0.0290 (5)	
C5	0.7453 (2)	−0.13655 (14)	0.5845 (2)	0.0287 (5)	
C6	0.6272 (2)	−0.13152 (14)	0.6261 (2)	0.0271 (5)	
C7	0.58787 (19)	−0.05499 (12)	0.6825 (2)	0.0223 (4)	
C8	0.46742 (19)	−0.04304 (13)	0.7273 (2)	0.0228 (4)	
C9	0.43492 (18)	0.03136 (12)	0.7863 (2)	0.0225 (4)	
C10	0.31072 (18)	0.04074 (13)	0.8374 (2)	0.0220 (4)	
C11	0.2471 (2)	0.12496 (14)	0.8465 (3)	0.0289 (5)	
C12	0.09542 (19)	−0.10427 (13)	0.9446 (2)	0.0241 (4)	
C13	0.1621 (2)	−0.18236 (13)	0.9000 (3)	0.0261 (5)	
C14	0.09896 (19)	−0.25873 (14)	0.9385 (2)	0.0267 (5)	
H3A	0.8460 (19)	0.0623 (14)	0.658 (2)	0.029 (6)*	
H4A	0.906 (2)	−0.0682 (13)	0.564 (2)	0.031 (6)*	
H6A	0.571 (2)	−0.1803 (14)	0.617 (2)	0.033 (6)*	
H5A	0.7711 (19)	−0.1885 (14)	0.543 (2)	0.030 (6)*	
H8A	0.4042 (19)	−0.0902 (13)	0.714 (2)	0.026 (5)*	
H11A	0.153 (2)	0.1204 (14)	0.794 (3)	0.035 (6)*	
H11B	0.284 (2)	0.1687 (15)	0.787 (3)	0.045 (7)*	
H11C	0.257 (3)	0.1423 (17)	0.966 (4)	0.075 (9)*	
H13A	0.255 (2)	−0.1847 (14)	0.962 (3)	0.040 (6)*	
H13B	0.155 (2)	−0.1803 (14)	0.777 (3)	0.043 (6)*	
H1N2	0.102 (2)	0.0198 (16)	0.941 (3)	0.049 (7)*	

### Atomic displacement parameters (Å^2^)

**Table:**

	*U*^11^	*U*^22^	*U*^33^	*U*^12^	*U*^13^	*U*^23^
O1	0.0218 (7)	0.0214 (8)	0.0357 (7)	0.0000 (6)	0.0159 (6)	−0.0019 (6)
O2	0.0266 (8)	0.0227 (8)	0.0483 (8)	0.0012 (6)	0.0160 (7)	−0.0072 (7)
O3	0.0265 (8)	0.0287 (9)	0.0423 (8)	−0.0002 (6)	0.0224 (6)	0.0001 (6)
N1	0.0211 (8)	0.0269 (10)	0.0284 (8)	−0.0005 (7)	0.0137 (7)	0.0006 (7)
N2	0.0215 (9)	0.0242 (10)	0.0340 (9)	0.0006 (8)	0.0161 (7)	0.0002 (7)
N3	0.0403 (11)	0.0299 (12)	0.0514 (11)	−0.0052 (9)	0.0246 (9)	−0.0030 (9)
C1	0.0232 (10)	0.0214 (11)	0.0302 (10)	0.0036 (8)	0.0104 (8)	0.0023 (8)
C2	0.0248 (10)	0.0186 (11)	0.0258 (9)	0.0048 (8)	0.0097 (8)	0.0000 (8)
C3	0.0241 (10)	0.0275 (13)	0.0313 (10)	−0.0003 (9)	0.0131 (8)	0.0005 (9)
C4	0.0231 (11)	0.0334 (13)	0.0348 (11)	0.0039 (9)	0.0153 (9)	−0.0005 (9)
C5	0.0317 (12)	0.0251 (12)	0.0331 (11)	0.0064 (10)	0.0152 (9)	−0.0009 (9)
C6	0.0319 (11)	0.0201 (12)	0.0329 (10)	0.0008 (9)	0.0154 (9)	0.0008 (9)
C7	0.0238 (10)	0.0199 (11)	0.0259 (9)	0.0023 (8)	0.0113 (8)	0.0015 (8)
C8	0.0252 (10)	0.0188 (11)	0.0278 (10)	−0.0016 (9)	0.0130 (8)	0.0019 (8)
C9	0.0220 (10)	0.0211 (11)	0.0272 (9)	0.0002 (8)	0.0116 (8)	0.0017 (8)
C10	0.0207 (10)	0.0241 (12)	0.0242 (9)	0.0009 (8)	0.0113 (8)	−0.0002 (8)
C11	0.0279 (12)	0.0255 (12)	0.0398 (12)	0.0032 (9)	0.0200 (10)	0.0034 (9)
C12	0.0226 (10)	0.0251 (12)	0.0277 (9)	0.0006 (9)	0.0118 (8)	−0.0004 (8)
C13	0.0252 (11)	0.0229 (12)	0.0357 (11)	−0.0032 (9)	0.0175 (9)	−0.0032 (9)
C14	0.0239 (10)	0.0265 (12)	0.0331 (10)	−0.0024 (9)	0.0137 (8)	−0.0049 (9)

### Geometric parameters (Å, º)

**Table:**

O1—C1	1.379 (2)	C5—C6	1.380 (3)
O1—C2	1.384 (2)	C5—H5A	0.96 (2)
O2—C1	1.209 (2)	C6—C7	1.402 (3)
O3—C12	1.225 (2)	C6—H6A	0.96 (2)
N1—C10	1.287 (2)	C7—C8	1.428 (3)
N1—N2	1.381 (2)	C8—C9	1.356 (3)
N2—C12	1.354 (2)	C8—H8A	0.98 (2)
N2—H1N2	0.96 (2)	C9—C10	1.488 (3)
N3—C14	1.133 (3)	C10—C11	1.502 (3)
C1—C9	1.469 (3)	C11—H11A	0.95 (2)
C2—C3	1.380 (3)	C11—H11B	0.99 (2)
C2—C7	1.396 (3)	C11—H11C	1.00 (3)
C3—C4	1.384 (3)	C12—C13	1.517 (3)
C3—H3A	1.00 (2)	C13—C14	1.457 (3)
C4—C5	1.390 (3)	C13—H13A	0.96 (2)
C4—H4A	0.96 (2)	C13—H13B	1.00 (2)
			
C1—O1—C2	123.02 (15)	C9—C8—C7	122.84 (19)
C10—N1—N2	118.20 (17)	C9—C8—H8A	117.9 (12)
C12—N2—N1	117.93 (17)	C7—C8—H8A	119.3 (12)
C12—N2—H1N2	114.9 (14)	C8—C9—C1	118.83 (18)
N1—N2—H1N2	126.7 (14)	C8—C9—C10	121.40 (18)
O2—C1—O1	116.01 (17)	C1—C9—C10	119.72 (17)
O2—C1—C9	126.87 (18)	N1—C10—C9	113.17 (17)
O1—C1—C9	117.12 (17)	N1—C10—C11	124.11 (18)
C3—C2—O1	117.10 (18)	C9—C10—C11	122.70 (18)
C3—C2—C7	122.69 (19)	C10—C11—H11A	108.8 (13)
O1—C2—C7	120.21 (17)	C10—C11—H11B	110.5 (13)
C2—C3—C4	118.0 (2)	H11A—C11—H11B	109.1 (18)
C2—C3—H3A	120.9 (12)	C10—C11—H11C	112.0 (16)
C4—C3—H3A	121.1 (12)	H11A—C11—H11C	105 (2)
C3—C4—C5	121.1 (2)	H11B—C11—H11C	111 (2)
C3—C4—H4A	118.5 (13)	O3—C12—N2	122.15 (18)
C5—C4—H4A	120.4 (13)	O3—C12—C13	122.03 (18)
C6—C5—C4	120.1 (2)	N2—C12—C13	115.81 (17)
C6—C5—H5A	120.3 (12)	C14—C13—C12	110.63 (16)
C4—C5—H5A	119.6 (12)	C14—C13—H13A	107.8 (13)
C5—C6—C7	120.3 (2)	C12—C13—H13A	111.6 (13)
C5—C6—H6A	120.7 (12)	C14—C13—H13B	110.2 (13)
C7—C6—H6A	119.0 (12)	C12—C13—H13B	108.4 (13)
C2—C7—C6	117.80 (18)	H13A—C13—H13B	108.2 (18)
C2—C7—C8	117.57 (18)	N3—C14—C13	178.3 (2)
C6—C7—C8	124.62 (19)		
			
C10—N1—N2—C12	179.26 (16)	C7—C8—C9—C1	−0.7 (3)
C2—O1—C1—O2	171.92 (16)	C7—C8—C9—C10	−177.89 (17)
C2—O1—C1—C9	−7.4 (2)	O2—C1—C9—C8	−173.41 (19)
C1—O1—C2—C3	−175.90 (16)	O1—C1—C9—C8	5.9 (3)
C1—O1—C2—C7	3.7 (2)	O2—C1—C9—C10	3.8 (3)
O1—C2—C3—C4	178.95 (16)	O1—C1—C9—C10	−176.90 (15)
C7—C2—C3—C4	−0.6 (3)	N2—N1—C10—C9	−179.11 (15)
C2—C3—C4—C5	−0.1 (3)	N2—N1—C10—C11	−0.8 (3)
C3—C4—C5—C6	1.0 (3)	C8—C9—C10—N1	19.6 (2)
C4—C5—C6—C7	−1.2 (3)	C1—C9—C10—N1	−157.52 (16)
C3—C2—C7—C6	0.4 (3)	C8—C9—C10—C11	−158.73 (18)
O1—C2—C7—C6	−179.18 (16)	C1—C9—C10—C11	24.1 (3)
C3—C2—C7—C8	−178.66 (17)	N1—N2—C12—O3	172.68 (16)
O1—C2—C7—C8	1.8 (3)	N1—N2—C12—C13	−8.4 (2)
C5—C6—C7—C2	0.5 (3)	O3—C12—C13—C14	−1.2 (3)
C5—C6—C7—C8	179.50 (18)	N2—C12—C13—C14	179.88 (16)
C2—C7—C8—C9	−3.2 (3)	C12—C13—C14—N3	−154 (7)
C6—C7—C8—C9	177.89 (18)		

### Hydrogen-bond geometry (Å, º)

**Table:**

*D*—H···*A*	*D*—H	H···*A*	*D*···*A*	*D*—H···*A*
N2—H1*N*2···O3^i^	0.96 (2)	1.91 (2)	2.870 (2)	174 (2)
C3—H3*A*···N3^ii^	1.00 (2)	2.53 (2)	3.446 (3)	152.9 (12)
C4—H4*A*···N3^iii^	0.96 (2)	2.62 (2)	3.404 (3)	139.2 (16)
C6—H6*A*···O2^iv^	0.96 (2)	2.56 (2)	3.494 (3)	164.7 (17)
C13—H13*A*···O2^v^	0.96 (2)	2.38 (2)	3.328 (3)	171.7 (18)
C13—H13*B*···N3^vi^	1.00 (2)	2.46 (2)	3.409 (3)	159.3 (18)

Symmetry codes: (i) −*x*, −*y*, −*z*+2; (ii) −*x*+1, *y*+1/2, −*z*+3/2; (iii) *x*+1, −*y*−1/2, *z*−1/2; (iv) −*x*+1, *y*−1/2, −*z*+3/2; (v) −*x*+1, −*y*, −*z*+2; (vi) *x*, −*y*−1/2, *z*−1/2.

## References

[bb1] Bruker (2009). *APEX2*, *SAINT* and *SADABS* Bruker AXS Inc., Madison, Wisconsin, USA.

[bb2] Sheldrick, G. M. (2008). *Acta Cryst.* A**64**, 112–122. 10.1107/S0108767307043930 18156677

[bb3] Spek, A. L. (2009). *Acta Cryst.* D**65**, 148–155. 10.1107/S090744490804362X PMC2631630 19171970

[bb4] Yusufzai, S. K., Osman, H., Abdul Rahim, A. S., Arshad, S. & Razak, I. A. (2012). *Acta Cryst.* E**68**, o1056–o1057. 10.1107/S1600536812009336 PMC3344016 22589925

[bb5] Yusufzai, S. K., Osman, H., Sulaiman, O., Arshad, S. & Razak, I. A. (2012). *Acta Cryst.* E**68**, o473–o474. 10.1107/S1600536812001432 PMC3275224 22347080

